# Synchronous Bilateral Breast Carcinoma and Axillary Non-Hodgkin Lymphoma: A Case Report and Review of the Literature

**DOI:** 10.1155/2012/685919

**Published:** 2012-09-23

**Authors:** Edward F. Miles, Laura L. Jacimore

**Affiliations:** ^1^Division of Radiation Oncology, Department of Radiology, Naval Medical Center Portsmouth, 620 John Paul Jones Circle, Portsmouth, VA 23708, USA; ^2^Department of Radiation Oncology, Nash General Hospital, 2460 Curtis Ellis Drive, Rocky Mount, NC 27804, USA

## Abstract

The use of staging imaging modalities with increased sensitivity has led to an increase in the incidence of detection of simultaneous malignancies. These cases require careful evaluation and discussion in a multidisciplinary setting to establish a treatment plan that optimizes the outcome with respect to each malignancy, particularly when treatment modalities overlap. We report a case of a patient diagnosed with axillary nodal diffuse large B-cell lymphoma (DLBCL) in a community hospital where staging workup also revealed synchronous bilateral breast carcinomas. To our knowledge, this is only the second case report of a patient with three synchronous primary malignancies: bilateral breast carcinomas and axillary DLBCL. The only other similar case report had no role for radiation or chemotherapy in the management of the indolent follicular lymphoma.

## 1. Introduction

Breast cancer constitutes approximately 14% of all primary cancers in women, accounting for more than 207,000 cases per year [1], but only a small fraction (2.2–4.3% in retrospective reviews) of these presented as bilateral breast cancer [2–4]. For early-stage disease, primary treatment involves surgical resection via modified radical mastectomy or breast conservation therapy with lumpectomy and sentinel lymph node biopsy. Adjuvant chemotherapy and hormonal therapy are often considered as well as completion of breast conservation therapy with radiation therapy to the involved breast and, if indicated, the regional lymph nodes [5]. Conversely, non-Hodgkin lymphoma constitutes only 4% of all primary cancers in women. Primary treatment for limited stage diffuse large B-cell lymphoma (DLBCL) involves systemic chemotherapy with or without consolidative radiation therapy, while late stage is treated primarily with systemic chemotherapy alone with radiation therapy considered for areas of original bulky disease [6]. 

Herein, we report a case of a patient diagnosed with axillary nodal DLBCL in a community hospital where staging workup also revealed synchronous bilateral breast carcinomas. A review of the relevant literature is also discussed. 

## 2. Case Report

**Figure 1:**
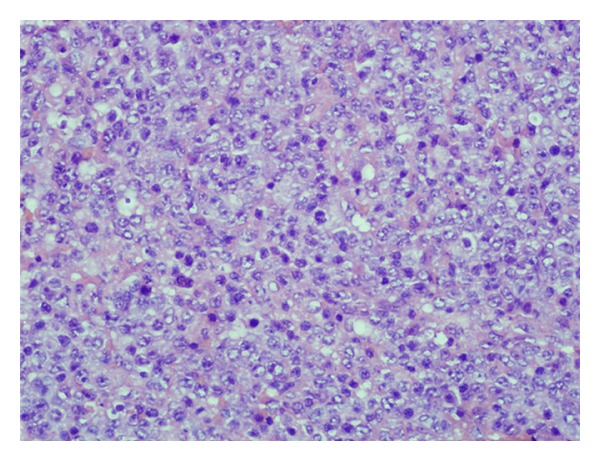
Right axillary node biopsy demonstrating diffuse large B-cell lymphoma.

**Figure 2:**
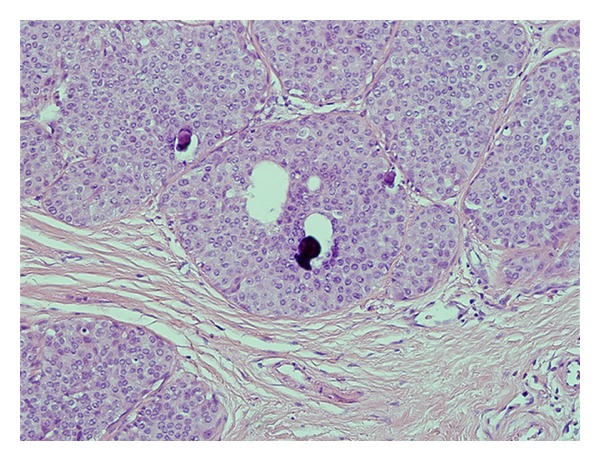
Right breast ductal carcinoma in situ.

**Figure 3:**
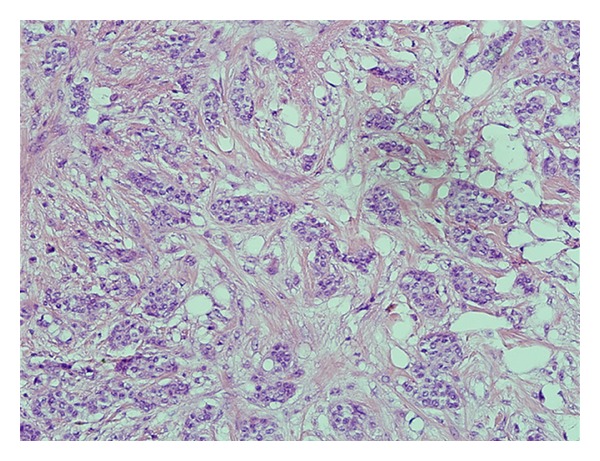
Left breast infiltrating ductal carcinoma.

**Figure 4:**
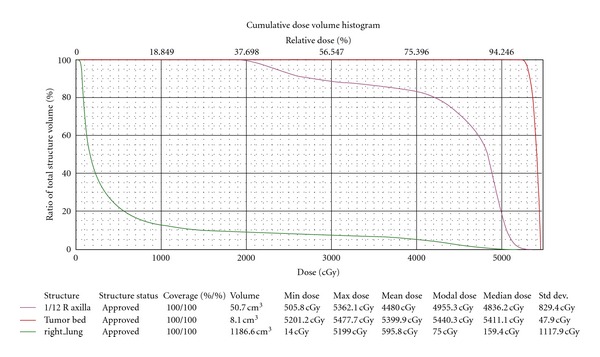
Dose volume histogram showing coverage of breast tumor bed (red) with standard breast tangents and incidental ipsilateral axillary coverage (purple).

The patient was a 64-year-old woman who presented to her local community hospital emergency department with a complaint of rapidly increasing edema to the right arm. She was noted to have significant right axillary adenopathy and although she denied fevers or night sweats, she had lost over 25 pounds (>10% of her body weight) in the three months prior to presentation. A computed tomography (CT) scan of the chest demonstrated multiple right axillary nodes and a questionable mass in the left breast. She underwent excisional biopsy of a right axillary node which demonstrated DLBCL (Figure 1). Her bone marrow biopsy was negative. Due to the findings on her staging CT scan and a palpable mass in the left breast, a bilateral mammogram was performed that showed a solid density in the vicinity of the left breast mass, but also showed calcifications in the medial right breast. Ultrasound-guided needle core biopsy of the breast lesions demonstrated ductal carcinoma in situ (DCIS) in the right breast (Figure 2), and estrogen and progesterone receptor positive, HER2 receptor negative infiltrating ductal carcinoma in the left breast (Figure 3). Due to issues with insurance and the relatively urgent need to treat her presumably Stage IB lymphoma, systemic chemotherapy with R-CHOP (rituximab, cyclophosphamide, adriamycin, vincristine, and prednisone) was started prior to a staging positron emission tomography (PET)/CT scan. Subsequent PET/CT scan showed a questionable area of “thickening” in the retrocrural area worrisome for lymphomatous involvement. She had a dramatic improvement in the right arm edema after the first cycle of chemotherapy. She continued systemic therapy for three cycles and then underwent a lumpectomy for the DCIS in the right breast (stage 0) and simple mastectomy with 2.1 cm of invasive disease with a negative sentinel lymph node biopsy on the left (stage IIa). Unfortunately, pathology revealed that the anterior margins for both resections were very close at less than 1 millimeter. Her case was discussed at a multidisciplinary tumor board, and a recommendation was made to reresect for clear margins bilaterally, and then due to the unfavorable risk factors for her DLBCL (age greater than 60 and B symptoms) and possible stage IIIB at diagnosis, to continue her systemic chemotherapy for an additional three cycles. PET/CT scan after completion of therapy revealed resolution of the retrocural thickening, indicating possible stage IIIB disease at diagnosis and two remaining PET-avid nodes in the right axilla. She underwent limited axillary dissection to determine her disease status and only necrotic tissue was found with no evidence of malignancy. Completion of her breast conservation therapy with standard breast tangent fields [7] allowed for incidental inclusion of levels I and II of the axilla in the treatment field with 88% of the right axilla receiving 3,000 cGy or more (Figure 4). With a mastectomy and negative sentinel lymph node biopsy for the left breast, local control therapy for this malignancy was considered complete. On the right, she had undergone breast conservation therapy, and her local therapy was completed with radiation therapy to the right breast. Routine followup has shown no clinical or radiographic evidence of any of her malignancies, and she continues on hormonal therapy for her receptor positive infiltrating ductal carcinoma.

## 3. Discussion

As the use of PET/CT scans for the staging of malignancies has become more common, the detection of second occult malignancies has also increased. A recent prospective study of non-Hodgkin lymphoma patients staged by PET/CT demonstrated that 2.9% of them had a second, occult, nonlymphoma malignancy [8]. Although there are well-established guidelines for the workup, staging, and treatment of individual malignancies, optimal treatment in the setting of multiple simultaneous malignancies is difficult. There are often competing urgency requirements, and the most pressing issue is treated first with an eye towards optimizing treatment for each malignancy as much as is possible. In this case, the rapidly expanding right arm edema secondary to her axillary lymphadenopathy from her DLBCL was this patients' most pressing issue. Per the National Comprehensive Cancer Network (NCCN) clinical practice guidelines, the standard of care for first-line treatment of Stage IB DLBCL in a patient with two adverse risk factors is systemic chemotherapy with R-CHOP for three cycles with involved field radiation therapy or six cycles with or without radiation therapy; if her retrocrural node was assumed to be positive, this would give her stage IIIB disease, and systemic chemotherapy for 6–8 cycles would have been indicated, with radiation considered to areas of initially bulky disease [6]. The systemic chemotherapy which was administered for treatment of the DLBCL also doubled as neoadjuvant and adjuvant therapy for the invasive ductal carcinoma, as three of the agents delivered (cyclophosphamide, doxorubicin, and vincristine) are active in breast cancer, and cyclophosphamide and doxorubicin are a standard regimen for resectable breast cancer. There is no role for systemic chemotherapy in DCIS. 

The literature contains several small case series and individual case reports of patients with primary breast malignancies with synchronous lymphoproliferative disorders including follicular lymphoma [9–11], small lymphocytic lymphoma/chronic lymphocytic leukemia [10, 12], Hodgkin lymphoma [10], mantle cell lymphoma [11], marginal zone lymphoma [13], and mucosa-associated lymphoid tissue (MALT) lymphoma [14, 15]. Two individual case reports document primary breast carcinoma with simultaneous DLBCL; these both occurred in the breast rather than in the axilla [16, 17]. The single case report that does discuss therapy indicates that six cycles of CHOP chemotherapy were administered as well as tamoxifen for hormone receptor positive invasive breast cancer. To our knowledge, this is only the second case report of a patient with three synchronous primary malignancies: bilateral breast carcinomas and axillary DLBCL. The only other case report had no role for radiation or chemotherapy in the management of the more indolent (follicular) lymphoma [13].

In conclusion, the appropriate use of sensitive staging studies makes the discovery of occult simultaneous malignancies a distinct possibility. Careful review of these studies with evaluation and discussion in a multidisciplinary setting ensures the most efficacious treatment regimen is planned and executed to maximize the chance of cure of these malignancies.
